# Doubly Resonant Optical Periodic Structure

**DOI:** 10.1038/srep20590

**Published:** 2016-02-08

**Authors:** G. Alagappan, C. E. Png

**Affiliations:** 1 Photonics and Plasmonics, Institute of High Performance Computing, Agency for Science, Technology, and Research (A-STAR), Singapore.

## Abstract

Periodic structures are well known in various branches of physics for their ability to provide a stopband. In this article, using optical periodic structures we showed that, when a second periodicity – very closed to the original periodicity is introduced, large number of states appears in the stopband corresponding to the first periodicity. In the limit where the two periods matches, we have a continuum of states, and the original stopband completely disappears. This intriguing phenomena is uncovered by noticing that, regardless of the proximities of the two periodicities, there is an array of spatial points where the dielectric functions corresponding to the two periodicities interfere destructively. These spatial points mimic photonic atoms by satisfying the standards equations of quantum harmonic oscillators, and exhibit lossless, atom-like dispersions.

Doubly resonant systems have compelling physical properties resulting from the interference effects. A three level atomic system with a Λ configuration has two resonant transitions, and by an appropriate coherent driving, we can generate steep positive (normal)^1^,^2^,^3^,^4^ and steep negative (anomalous)^5^,^6^,^7^ optical dispersions. Such steep dispersions can be exploited to create novel systems of slow light and subluminal light without any violation in the Einstein’s causality principle^8^,^9^. Steep positive dispersions are usually obtained in the three level systems using an electromagnetic induced transparency (EIT) setup^1^,^2^,^3^, and there have been many proposals to mimic such configuration using plasmonic^10^,^11^ and optical^12^,^13^,^14^,^15^ double resonances. These mock versions work based on the coherent interference effects in the light scattering, and allow tuning of the positive dispersions via modifications in the geometrical structures. They also have been shown to possess scattering dark states^16^ and superscattering states^17^,^18^. In this article, we demonstrate the intriguing optical properties of a new paradigm of doubly resonant systems that exploits structures with both short and long range spatial periodicities, and exhibiting two closely spaced Bragg resonances.

Typical periodic structures are single – period, structures (SPSs), and they exhibit stopbands [i.e., spectral regions for which wave propagations are forbidden]. The physical principle behind this stopband formation is the Bragg resonance of the SPS. Waves with frequency in the vicinity of Bragg resonance frequency, will experience a strong Bragg reflectivity, and therefore is unable to penetrate the bulk of the SPS.

In optics a SPS can be created by mean of a periodic variation of the dielectric constant with a fixed spatial period, *a*. We can again modulate the dielectric profile of this SPS, slowly and periodically, with a longer spatial period, *a*_*s*_^19^,^20^,^21^. This new periodic structure which exhibits rapid, short range periodicity (*a*) and slow, long range periodicity (*a*_*s*_) is defined as a dual periodic structure (DPS). Intuitively, one can expect in the limit of a very large *a*_*s*_, the slow modulation vanishes, and consequently a DPS reduces to a SPS. However, a DPS in this limit does not fit into this simple intuition.

In the Fourier spectra, the dielectric function of a weakly modulated SPS, will exhibit one frequency peak at the fundamental spatial frequency *G* = 2π/*a*. However, for the DPS, due to the slow dielectric modulation, we will see a group of closely spaced peaks around the fundamental frequency. Assuming a DPS with only two of such closely spaced peaks (i.e., a structure with double Bragg resonances), the dielectric function can be casted as,


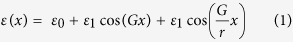


where *r* is a number close to 1. In Eqn. 1, 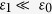, and for a simplicity, we assumed the strengths of the two closely spaced harmonics to be equal (i.e., the amplitudes of two cosine functions in Eqn. 1 are equal). The conservation of translational symmetry in DPS requires 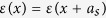, and using Eqn. 1 it can be shown that this demands 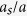 to be the least integer multiple of *r*. The least integer multiple exist, only if *r* is rational. Assuming a rational *r*, and taking its’ least integer multiple as *R*, we have 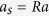.

In a DPS, the mixing of the two harmonics, *G* and *G*/*r* creates the spatial “beats” in the dielectric function at a longer spatial scale. The length of the beat (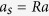) is longer when the spacing between the two harmonics is closer. The closest allowed proximity between the two harmonics, *G* and *G*/*r* is one reciprocal lattice vector of the DPS, *g* = 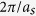. Any spacing lesser than *g*, is symmetrically forbidden, and hence will break the translational symmetry of the DPS. Assuming 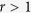, and the spacing, *G* − *G*/*r* = *g*, it is easy to show that the rational form of *r* is *r* = *R*/(*R* −  1) [or equivalently *R* = *r*/(*r* − 1)].

For a SPS, *r* = 1, and from Eqn. 1 we have 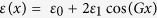. For the DPS, a direct substitution of 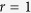 for the limit 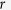 → 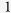 in Eqn. 1 leads to a plausible inference that the DPS should be identical to the SPS in the limit 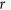 → 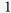, and therefore recovers the original stopband of the SPS. However, the constraint *r* = *R*/(*R* − 1) for the DPS prevents the direct substitution of 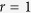 for the limit 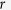 → 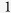 in Eqn. 1. The flawless method of analysing the limit 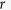 → 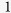 in DPS is by letting *R* to take a huge integer value. As an illustration, Fig. 1(a) shows a sketch of 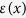 with the unit cell from 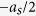 to 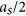 is highlighted in blue. Figure 1(b) depicts the evolution of the unit cell as *R* is increased to a huge integer value. As we can see from Fig. 1(a,b), regardless of the proximity of *r* to 1, the dielectric function of the DPS is topologically different from the dielectric function of the SPS – which is an unmodulated cosine function. As a signature difference, in DPS, the destructive interference between the two cosine waves in Eqn. 1 creates an array of spatial points (i.e., the green dots in Fig. 1) that are shielded from the effect of the rapid dielectric modulation with the period *a*. As we shall illustrate, this array of spatial points mimics an array of photonic atoms by satisfying the standard equations of quantum harmonic oscillators^22^. These photonic atoms create edge states (at the edge of the DPS unit cell) that closes the stopband due to the rapid dielectric modulation, despite the limit 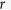 → 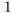. The dispersions in the vicinity of these photonic atoms, are strongly anomalous (i.e., a steep negative dispersion), and very much similar to the dispersions in mediums with inverted populations^23^,^24^ and gain doublets^5^,^6^,^7^.

## DPS as a Metamaterial Cavity

For the purpose of the numerical illustration, throughout this article, we use 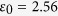, and 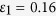. An optical structure with such dielectric constants, and the dielectric profile as in Eqn. 1 with a large *R*, can be realized in many different ways. Some of the techniques include, fabrication of porous silicon via electrochemical anodization with varying current density^25^, deposition of a dual periodic multilayer using a logical combination technique^19^, holographic interferometry, that make uses laser beam interferences on photosensitive materials^26^, and the deposition of silicon oxynitride with varying stoichiometry of oxygen and nitrogen^27^,^28^.

Using a plane wave expansion method^29^, we can solve the dispersion relation, 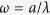 versus *k*, where 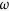, 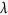 and *k* are the normalized frequency, freespace wavelength and wavevector, respectively. Firstly, consider the limiting case, when *r* → 1. As *r* = *R*/(*R* − 1), the dispersion curve of the DPS in this limit can be obtained asymptotically by increasing *R* to a huge integer value, using an extended zone scheme^30^. For a very large *R*, the dispersion of the DPS converges to a continuous curve, 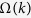, shown in Fig. 2(a) [blue curve]. In the same diagram, we have also plotted the dispersion curves of the SPS, and a homogenous medium with dielectric constant 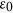. When *r* → 1, from the direct substitution of *r* = 1 in Eqn. 1, one can expect the dispersion of the DPS to be identical to the dispersion of the SPS. However, this is only true for wavevectors far from *G*/2. For wavevectors far from *G*/2, both SPS and DPS behave as linear homogenous materials of dielectric constants 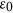. Near *k* = *G*/2, the dispersion relation of the DPS is remarkably different from the dispersion relation of the SPS despite *r* → 1 [Fig. 2(a)]. In the SPS, the dispersion is discontinuous at *k* = *G*/2, and consequently we have a stopband between the frequencies 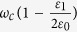 and 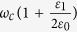, where 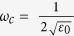 is the stopband centre^29^. In the DPS this stopband completely closes, and the dispersion relation takes a continuous, sigmoid shape curve around 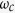. Thus, in the vicinity of 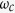, the DPS with *r* → 1, can be characterized with a dispersive refractive index, 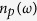. This refractive index can be defined via the phase index definition as 
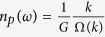
, and the corresponding plot as a function of frequency is shown in Fig. 2(b). From this figure, we can see that 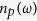 exhibits a large normal dispersion (i.e., a positive 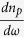) near the stopband edges of the SPS. However, near 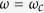 the dispersion is strongly anomalous (i.e., negative 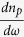). In order to better quantify the dispersion of the DPS with *r* → 1, let’s define a group index via 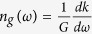. Note that 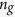 is inversely proportional to the slope of the dispersion curve 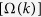, and its relationship with 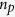 can be casted as 
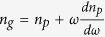
. Figure 2(c) shows the plot of 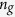 as a function of frequency, and as we can see from this figure 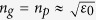 for frequencies far away from the two SPS stopband edges. For frequencies near the stopband edges of the SPS, 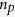 exhibits a large normal dispersion [Fig. 2(b)], and consequently we have a subluminal group index with 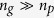 [Fig. 2(c)]. On the other hand, near 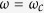 the 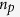
exhibits a large anomalous dispersion, and consequently this gives a superluminal group index 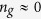 [Fig. 2(c)]. The anomalous dispersion, and the resulting superluminal group index of the DPS in the limit *r* → 1, is very much similar to anomalous dispersions in the nonlinear mediums with population inversions^23^,^24^, and gain doublets^5^,^6^,^7^. Besides these active nonlinear structures, anomalous dispersion has been also shown as a result of scattering in passive structures that facilitates tunnelling of light^8^,^9^,^31^,^32^,^33^,^34^. However, such system is very lossy since the anomalous dispersion is for the evanescent solution of the system. On the other hand, in the case of DPS (with the limit *r* → 1), the anomalous dispersion is for the real solutions (i.e., propagating waves) of the system.

The refractive indices (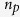 and 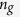) obtained for the limit *r* → 1 is indeed useful to describe and infer the behaviour of the DPSs with finite values of *R*. For a finite *R*, the DPS behaves like a multimode optical cavity made of an artificial material with the dispersive refractive index 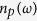. For an illustration, in Fig. 3(a), we show the dispersion curve for *R* = 500. As we can see from this figure, the dispersion curve for *R* = 500, which is in similar shape as 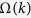 [Fig. 2(a)], is discontinuous at each half of the Brillouin zone (BZ), forming discrete bands in the vicinity of 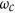. Imporatanltly, these discrete bands are flat, signifying the dispersions of slow or localized optical modes^35^. The dispersion curve for *R* = 500 in the reduced zone scheme is shown in Fig. 3(b). Note that, the dispersion curve in the reduced zone scheme is better known in the name of photonic band structure^29^,^36^. In Fig. 3(c–f), we show the photonic band structures for *R* =750, 1000, 2000, and 5000. The frequency positions of the flat bands, to a very good approximation is given by


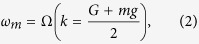


where *m* is a integer that indexes the flat band in the photonic band structure. The indexing scheme is shown in Fig. 3(b). The frequency spacing between the flat bands, 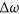, can be expressed using the group index of the DPS in the limit *r* → 1 [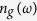] as


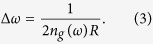


This frequency spacing for the DPS with the finite *R* is the same as the frequency spacing in a Fabry Perot cavity made with a dispersive dielectric material of refractive index 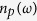, with a length *Ra*. From Fig. 3(b–f), we can see that the density of the flat bands increases as a function of *R*. This is because the discretization step (i.e., the length of half BZ 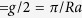) decreases as *R* increases. Further, these figures also indicate that the density of flat bands are not uniform across the frequencies of the stopband. For each *R* in Fig. 2(b–f), the density of the flat bands is maximum near SPS band edge frequencies, and the density is minimum near the SPS stopband centre. These density variations are due the dispersive nature of 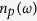, and can be easily understood from the frequency spacing [Eqn. 3] which is inversely proportional to 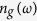. The values of 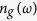 are maximum and minimum for SPS stopband edges and stopband centre, respectively [see the 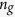 plot in Fig. 2(c)]. Therefore, the densities of flat bands are maximum and minimum for frequencies near SPS stopband edges and stopband centre, respectively.

## DPS as a Photonic Harmonic Oscillator

**Figure 1:**
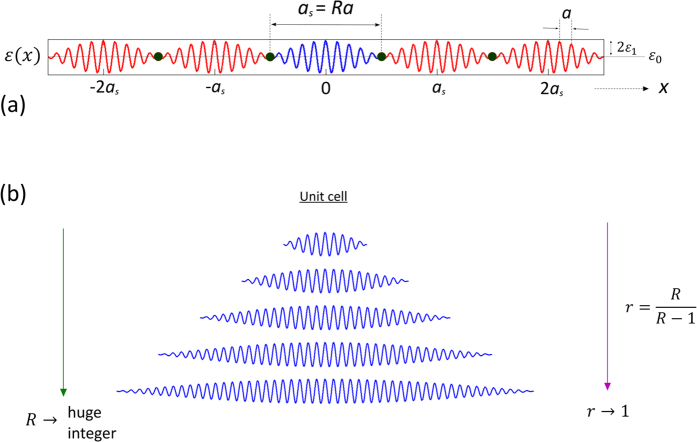
(**a**) Sketch of Eqn. 1 with *r* = *R*/(*R* − 1). At the location of green dots we have 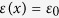. (**b**) Evolution of the DPS unit cell as a function of increasing *R*.

In order to perceive the intriguing dispersion of the DPS, let us examine the dielectric function in the vicinity of green dots in Fig. 1. For the sake of discussion let us pick *x* = *a*_*s*_/2. Using the approximation 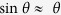 for a small angle 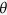, it can be easily shown that near *x* = *a*_*s*_/2, and for a large *R* Eqn. 1 becomes 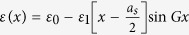. As can be seen from this equation, in the proximity of *x* = *a*_*s*_/2, the strength of the rapid dielectric modulation–the modulation with the period *a* (i.e., the amplitude of sin *Gx*) is 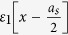, and it is a linear function of 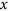.

When 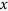 is exactly 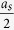, the strength of the rapid dielectric modulation is zero, and we have 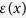 = 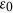. This means, at this position the DPS is completely shielded from the effect of the SPS (i.e., the rapid dielectric modulation). So, any light with a frequency in the vicinity of the SPS stopband centre (i.e., 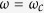) tends to concentrate at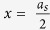. A slight deviation from 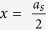, causes the light to face the rapid dielectric modulation of the SPS in a linearly increasing strength, 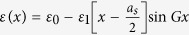, and as a consequence the light will be reflected back towards 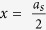. As the stopband resulting from the rapid dielectric modulation, is proportional to the strength of the modulation 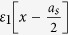, the reflection will be stronger as the deviation from 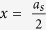 increases. As we shall prove in the following, this scenario is analogous a lossless harmonic oscillator with linearly increasing restoring force^30^.

In order to analyse the localized modes at 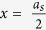, let us first move the origin of the 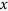
–axis from 0 to 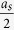. In the new coordinate system, Eqn. 1 becomes 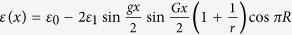. Near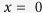, assuming an even *R*, we have 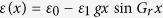, where 
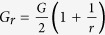
. In Fig. 4(a), we illustrate 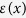, and its slowly varying amplitude functions in the new coordinate system. As we can readily see from this figure, 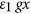 provides a very good approximation to the slowly varying amplitude over half of the unit cell [i.e., from −*a*_*s*_/4 to *a*_*s*_/4]. With 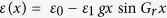, the time–independent Maxwell’s equation for the light in the DPS can be written as,


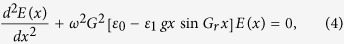


where 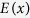 is the electric field. In order to solve Eqn. 4, assume the DPS to be a SPS with a linearly perturbed dielectric function in the slow spatial scale. Consequently, 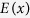 can be expressed as a linear combination of the SPS’s modes,


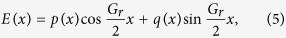


where 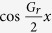 and 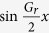 are the modes of the SPS which are rapidly varying functions. The coefficients 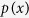 and 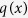 are slowly varying functions. Substituting Eqn. 5 into Eqn. 4, and applying a slowly varying envelope approximation^37^, we can average out the rapidly varying terms. The resulting slow scale equations are coupled equations,


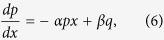



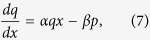


where 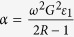, and 
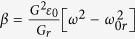
 are frequency dependant constants. Here,


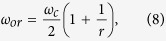


with 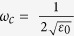 is the center of SPS bandgap. Changing the variable, 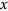, to a dimensionless position variable, 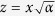, and eliminaing *q* and *p* in Eqns 6 and 7, respectively, we have two independent second order differential equations,


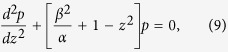



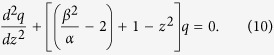


Eqns 9,10 mimic the standard Schrodinger equation for a quantum harmonic oscillator, 
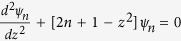
 with *n* being a non–negative integer. The solution for the Schrodinger equation is 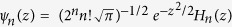, where 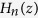 is the Hermite polynomial of order *n*^38^. Therefore, to solve Eqns 9,10, we let 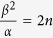, and subsitituting the frequency expressions for 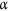 and 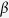, gives us,


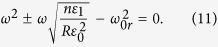


Assuming 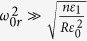, the positive solutions to Eqn. 11 are 
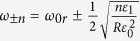
 . In order, to be consistent with the frequency indexing scheme used in Fig. 3(b), this solution also can re-written for any integer *m* as,


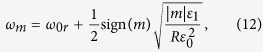


where 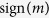 > 0 and 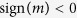 for positive and negative values of *m*, respectively.

As we have let, 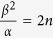 in Eqn. 9, we have 
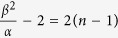
 for Eqn. 10. Thus, for a non–negative 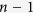, we need 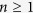, and consequently for *n* = 0 we have 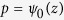 and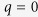. For 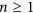, the solutions for 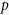 and 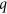 in Eqns 9,10 can be written as 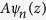, and 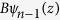, respectively. Here, *A* and *B* are constants, and using Eqns 6 and 11, it can be shown that they satisfy 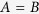 for 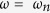, and 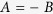 for 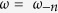. With the solutions for 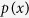 and 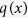, and the normalization condition 
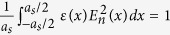
, the electric fields [Eqn. 5] for the modes with frequencies 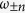 can be succinctly written as


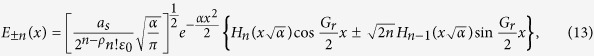


where 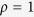 and 0, for 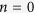 and 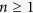, respectively. As 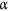 is real and positive, the modes described by Eqn. 13 exhibit Gaussian evanescent tails, 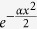, which decay smoothly.

Eqn. 13 also can be written as 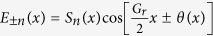, where 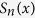 and 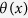 are the slowly varying amplitude and phase, respectively. From Eqn. 13, we can show that,


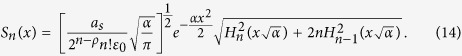


For *n* = 0, 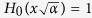, and we have Gaussian function for 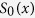. In Fig. 4(b) we plot 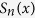 using Eqn. 14 for *n* = 0 to 5, and *R* = 750. In the same figure, we have also plotted the similar quantity obtained from the exact numerical calculation (based on the plane wave expansion method^29^. Figure 4(c) shows a similar plot to Fig. 4(b), however for *R* = 2000. As we can see from these figures, both analytical and exact calculations are in very good agreement. The degree of agreement reduces when the slowly varying electric field amplitude moves far from the centre of the DPS’s unit cell.

In order to determine the continuous dispersion relation in the limit *r* → 1, let us write the wavevector in the extended zone scheme as 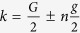. Using this *k*, and the frequency expression, 
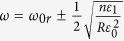
, it can be shown that, in the vicinity of 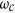 and *r* → 1, the dispersion curve of the DPS is defined by


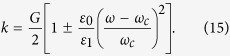


**Figure 2:**
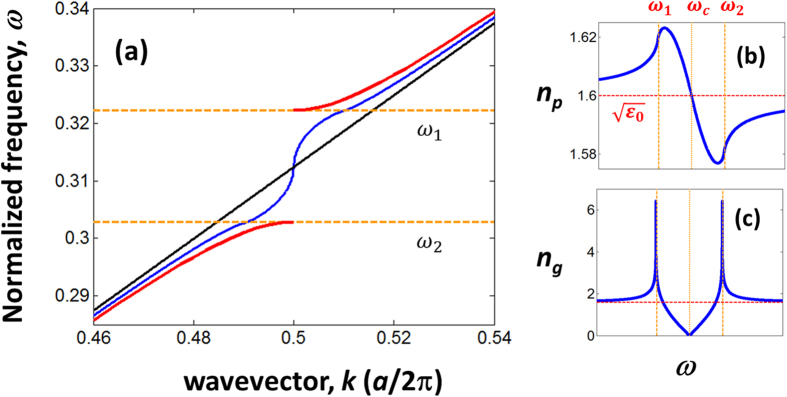
(**a**) Dispersion curves. Black – Homogenous medium with the dielectric constant 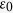, Red – SPS, Blue – DPS in the limit 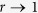. (**b**) Refractive index, 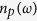 for the DPS in the limit 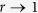. (**c**) Group index 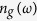 for the DPS in the limit 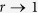. In (**b,c**), the dashed vertical lines represent the SPS stopband edges (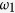 and 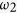), and the stopband centre (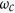). The dashed horizontal lines in (**b,c**) correspond to the refractive index, 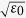.

In Fig. 4(d), we compare the dispersion curve obtained from Eqn. 15, with respect to the exact numerical calculation (i.e., the blue curve in Fig. 2). As we can see from this figure, we have a good agreement between these two curves for frequencies in the stopband of the SPS, and in the vicinity of 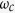, the agreement is perfect. The discrepancies in the wavevector values of the analytically obtained curve with respect to the numerically evaluated curve are less than 0.01% and 1% for frequencies near 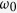 and near the stopband edge, respectively. From Eqn. 15, the closed form expression of the refractive index is 
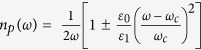
. The group index can be obtained by differentiating Eqn. 15 with respect to 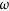 as 
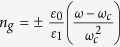
. As we can see from this expression, for 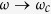, we have 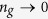. This zero group index signifies that the light propagation in the vicinity of the equilibrium position [*x* = 0 (new coordinate system) in Fig. 4(a); green dot in Fig. 1(a)] is essentially a tunnelling process, and therefore superluminal in nature^8^,^9^,^31^,^32^,^33^,^34^.

## Application and Optical Performances of DPS

DPSs can be used for designing high quality broadband, and multichannel slow light devices. The harmonic modes of the DPS exhibit Gaussian evanescent tails, which decay smoothly. Therefore it naturally generates resonant peaks of high quality factors^39^, despite of a geometrical structure with a low refractive index contrast. This is favourable for many applications such as high temperature realization of Bose-Einstein condensation of exciton polaritons^40^, and realization low threshold nonlinear optical devices, using the abundant low refractive index optical materials.

**Figure 3:**
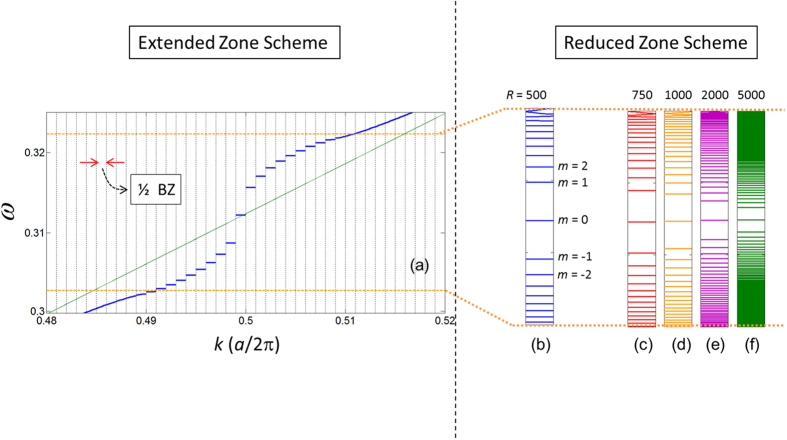
(**a**) Blue – dispersion curve of the DPS with *R* = 500 in the extended zone scheme. Green – dispersion curve of a homogenous medium. (**b–f**) Dispersion curves in the reduced zone scheme (i.e., photonic band structure) for *R* = 500, 750, 1000, 2000, and 5000. The horizontal axes in (**b–f**) represent wavevectors in the half of the BZ. The photonic band structure is obtained by folding the dispersion curve in the extended zone scheme into the half of the BZ. The dashed orange lines indicate the band edges of the SPS. The label *m* in (**b**) represents the frequency subscript as in 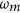.

In the metamaterial cavity section, we showed that for a finite *R*, the DPS exhibits many flat bands (for example see Fig. 3). In the transmission spectrum, these flat bands will appear as sharp resonant peaks. Figure 5(a) shows the schematic of a single unit cell DPS (*N* = 1). This single unit cell DPS acts as a metamaterial cavity. The dielectric constant of the ambience is taken as 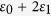 to match with the dielectric constant at the edge of the unit cell [Fig. 5(a)]. The device with the schematic as shown in Fig. 5(a) can be easily fabricated using the holographic interferometric techniques^26^. Figure 5(b) shows the simulated transmission spectrums [see the Methods section for the details of the numerical simulation] of the single unit cell DPS for *R* = 100, 200, 300, and 400. The frequency window in this figures spans the entire bandgap window of the SPS [i.e., 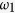 to 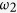; see Fig. 2(a)]. As we can from this figure, the transmission spectrums of the DPS exhibit many sharp resonant peaks, and the number of peaks increases as *R* increases. The density of the transmission peaks are high and low near the bandgap edge and bandgap centre of the SPS, respectively. These observations are consistent with the band structure calculations [see section on the metamaterial cavity]. The density of the transmission peaks also agrees with the equation describing the frequency spacing of the flat bands [Eqn. 3].

Each sharp transmission peak in Fig. 5(b), can be labelled with an integer *m* using the frequency indexing scheme used in Fig. 3(b). We showed the frequency labelling for the transmission peaks of *R* = 400 in Fig. 5(b). This labelling will assist us to compare the locations of simulated transmission peaks with the theory developed in this paper. Figure 5(c) compares the positions of the transmission peaks with the resonant frequencies obtained from Eqn. 2 [i.e., from the continuous dispersion curve: blue curve in Fig. 2(a)] and Eqn. 12 [i.e., from the theory of photonic harmonic oscillators]. As the figure suggests, both of these frequency expressions serve as good approximations to the frequencies of the transmission peaks.

The most profound transmission peak of the DPS is the peak with *m* = 0. This peak has the highest quality factor, and the peak is visible even for small values of *R*. In Fig. 5(d), we plotted the frequency of the *m* = 0 resonant transmission peak (obtained with the full numerical simulation; see the Methods section) as a function of *R*. As *R* increases the frequency of the *m* = 0 peak move towards to the centre of the SPS bandgap, 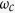. The position of the *m* = 0 transmission peak is in perfect agreement with the prediction of the harmonic oscillator theory which gives 
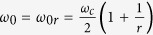
 [Eqns 8 and 12].The plot 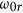 is shown in purple color in Fig. 5(d) for the continuous scale of *R*. As 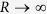, we have 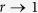, and therefore 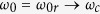. In order to assess the quality of transmission peaks with *m* = 0, in Fig. 5(e), we plot the logarithmic values of the their quality factors as a function of *R*, for 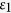 values of 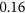 and 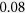. As we can see from this figure, the quality factor increases exponentially as *R* increases, and the quality factor is comparatively high when the dielectric modulation is of the DPS is high.

For *N* > 1 the finite DPS is indeed a coupled system of harmonic oscillators [Fig. 1]. An essential nature of any coupled oscillators is the splitting of the resonant peaks^41^,^42^. The DPS with *N* > 1, and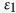 = 0.16 [the schematic is shown in Fig. 1] is numerically simulated using an ambience dielectric constant of 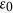 [see the Methods section for the details]. Figure 6(a) shows the resulting transmission spectrum near the frequency 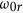 for *N* = 2, 3, 4 and 5. When *N* = 2, there is one transmission peak with frequency of 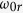. This peaks corresponds to the localized mode of *m* = 0, in the vicinity of one green dot in Fig. 1. When *N* = 3, there are two of such modes couples together, and as a result the original peak at *N* = 2 splits in two peaks [see Fig. 6(a)]. In general, for the DPS with *N* unit cells, the coupling of modes in adjacent oscillators results in *N*-1 closely spaced peaks. The splitting of the peaks is systematically depicted in Fig. 6(b). The frequency span of these closely spaced peaks equal to the bandwidth of the *m* = 0 flat band of the infinite system (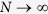), and the span can be obtained from the DPS band structure calculations. For *R* = 50, and 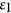 = 0.16 the frequency span obtained from the band structure calculation is from 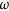 = 0.309 to 0.3098. If we choose to work around the telecommunication wavelength, then, for *a* = 480 nm, this normalized frequencies translates to wavelengths from 1549 to 1553 nms. This frequency span is adjustable. If we would like to have a narrow frequency span, then *R* has to be increased to generate a flatter band.

The closely spaced peaks for any given *N* in Fig. 6(a) display non-uniform quality factors. The outermost peak [see Fig. 6(a,b)] exhibits the largest quality factor. In Fig. 6(c), we show the quality factor of the outermost peak [i.e., along the line *A* - insert of Fig. 6(c)] as a function of *N*. As *N* increases, the quality factors increases quadratically. Note this increase of quality factor with respect *N* is slower than the increase of quality factor with respect *R*, which is at the exponential scale [Fig. 5(e)].

## Constructing a high dielectric contrast DPS

As we have mentioned in the metamaterial cavity section, the DPS with the dielectric function as in Eqn. 1 can be implemented in many different ways^25^,^26^,^27^,^28^. Most of these methods generates continuous dielectric profile, and therefore they have two important limitations: 1) generating structures with large dielectric modulations (i.e., large 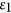); 2) generating structures that are amenable for mass production via lithographical techniques. Therefore, in this section, we would like to introduce the method of creating dielectric profiles as in Eqn. 1, however, with a large dielectric contrast. The method can be implemented either using a multilayer deposition or standard lithographical techniques.

Recall that Eqn. 1 is actually a cosine series. Therefore, in general, we will have a DPS as long as the Fourier series of any periodic dielectric function, at least in an approximation, takes the form of Eqn. 1. Thus, what is really needed to form a DPS, is a dielectric function with two closely spaced frequency peaks (at frequencies *G* and *G*/*r*) in its’ spatial Fourier spectra.

Consider a dielectric profile, 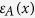 as in Fig. 7(a) which has a period *a*. This is a binary profile with alternating dielectric constants of 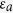 and 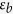. The fundamental harmonic of 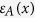 occurs at the frequency *G* = 2π/*a*. In Fig. 7(b), we have a similar dielectric function 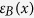, but with a period *ra*. The fundamental harmonic 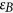
(*x*) is therefore, at the frequency 2π/*ra* = *G*/*r*. Now if we linearly combine 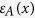 and 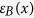 as 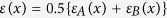 [see Fig. 7(c)] then by the linearity of the Fourier transform, the new function 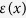 will have two Fourier peaks at frequencies *G* and *G*/*r*. Therefore to a good approximation this results in dielectric profile similar to Eqn. 1. Note that in Fig. 7(a,b), the dielectric functions are binary valued, however the dielectric function in Fig. 7(c) is not a binary profile. In order to implement this dielectric profile, we need three materials with dielectric constants 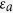, 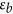, and 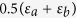.

Although the linear combination looks simple in its’ operation, the real implementation requires a third material with the dielectric constant 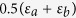. This condition can be relaxed, if we use a logical combination, instead of the linear combination. The output of a logical combination is always binary, and therefore if we combined 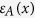 and 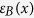, using a logical operation at every *x*, then we will obtain a dielectric profile with the binaries 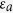 and 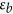. Further, if we propely choose the duty cycle of 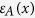 and 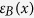, this will also give two strong harmonics at frequencies *G* and *G*/*r*. For an example in Fig. 7(a,b), assume the length of the 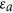 portion within each period is 0.2*a*. If we treat 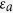 and 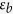 to be equivalent to the binaries 1 and 0, and logically combine, 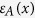 and 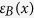, using a logical OR combination, then the result is the dielectric profile as shown in Fig. 7(d). The Fourier transform of this dielectric profile is shown in Fig. 7(e) for 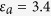 (silicon) and 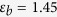 (silicon dioxide). As we can clearly see, the Fourier transform exhibits two profound peaks at the frequencies *G* and *G*/*r*.

Therefore, using the method of linear and logical combinations, we can generate non-continuous, high dielectric contrast DPS structures. The dielectric profiles in Fig. 7(c,d) can be easily fabricated either via multilayer deposition, or lithographical techniques. One important point to note when handing high dielectric contrast logically or linearly combined structures is that the their Fourier transforms will also consists the higher order harmonics. Nevertheless, the continuous dispersion curve for the limit 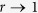 is still obtainnable via exact numerical calculations, and the photonic harmonic oscilator theory will serve as a qualitative model that gives a good physical perspective.

## Conclusion

**Figure 4:**
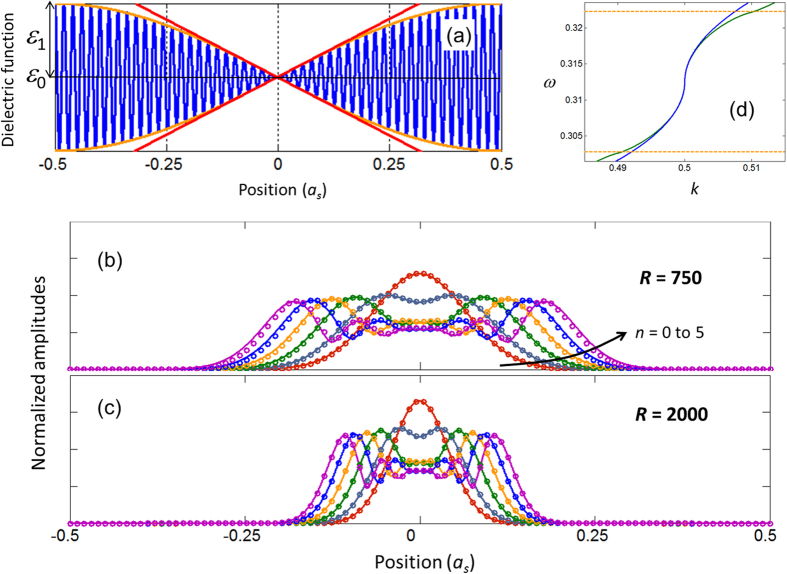
(**a**) Blue – the dielectric function of the DPS, 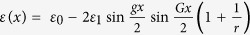. Orange – the slowly varying amplitude of 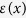, 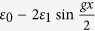. Red – the slowly varying amplitude of 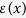 near 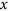 = 0, 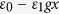. (**b,c**) Normalized electric field amplitudes for the localized modes (*n* = 0 to 5) of the DPS. Circles – analytical calculations via Eqn. 14. Solid line – exact numerical calculations. (**d**) The dispersion curves in the limit *r* → 1. Blue – analytical calculation via Eqn. 15. Green – exact numerical calculation (same as the blue curve in Fig. 2).

**Figure 5:**
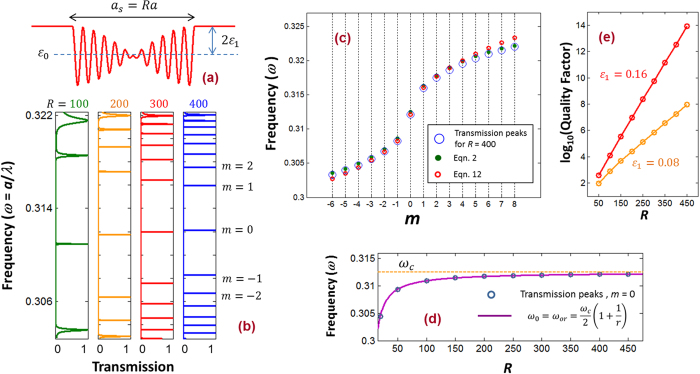
(**a**) Schematic of the single unit cell DPS. (**b**) Transmission spectrums for *R* = 100, 200, 300 and 400. (**c**) (blue-open circles) Frequencies of transmission peaks for *R* = 400 from “(**b**)”, and frequencies from Eqn. 2 (green-closed circles), and Eqn. 12 (red-open circles). (**d**) Frequencies of *m* = 0 transmission peaks obtained from full numerical simulations (blue circles), and frequencies from the harmonic oscillator theory for *m* = 0 (solid purple line). (**e**) logarithmic value of the quality factors for *m* = 0 peaks as a function of *R*.

**Figure 6:**
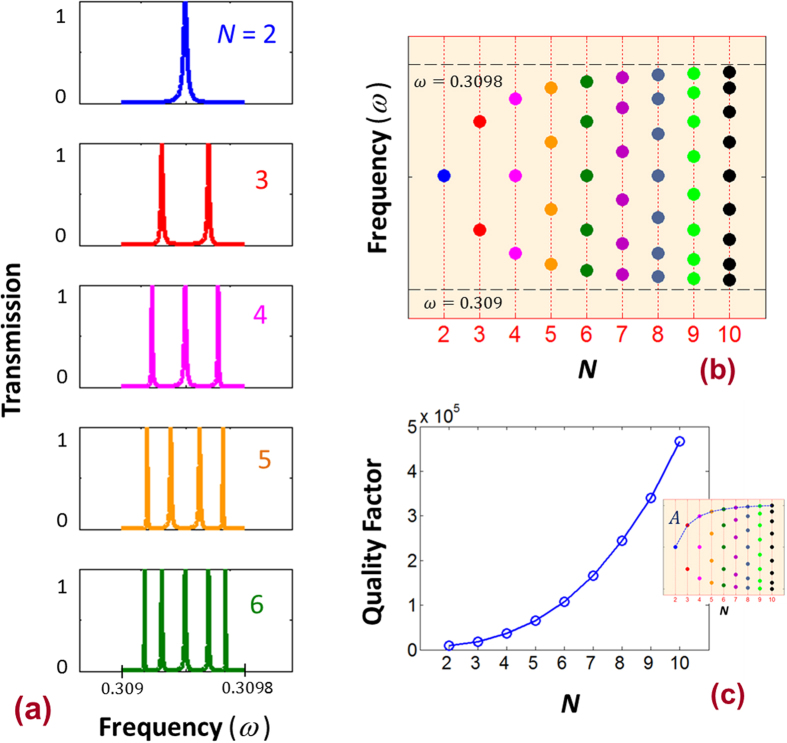
(**a**) Transmission peaks of a DPS (*R* = 50) with *N* unit cells that corresponds to *m* = 0 flat band (**b**) Frequencies of *m* = 0 flat band as a function of *N*. The *m* = 0 flat band has a frequency span of 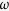 = 0.309 to 0.3098. (**c**) The quality factors of the transmission peaks along the path *A* (see the insert).

**Figure 7:**
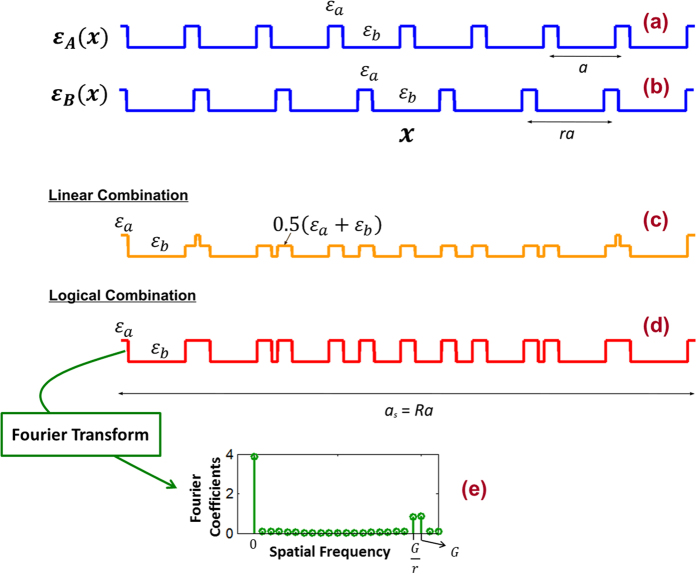
(**a**) Binary valued periodic dielectric function [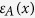] with period, *a*. (**b**) Binary valued periodic dielectric function [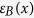] with period, *ra*. (**c**) Linear combination of 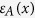 and 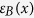,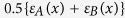. (**d**) Logical “OR” combination of 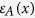 and 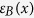 [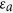 and 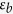 are treated as binaries 1 and 0, respectively] (**e**) Fourier transform of the periodic dielectric function (period = *a*_*s*_) in “(d)”. Here, we assumed 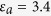 (silicon), 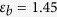 (silicon dioxide), and the length of the 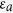 portion within each period of 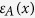 and 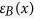 as 0.2*a*.

In conclusion, we have presented the unique dispersion properties of a DPS with two closely spaced harmonics. Our discussion confirms that the anomalous dispersion of the DPS in the vicinity of 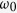, is due to the linear perturbation in the dielectric function of the SPS. As we have shown, this linear perturbation is analogous to a presence of a harmonic oscillator, which pulls the light back towards the equilibrium position [i.e., *x* = 0 in Fig. 4(a), new coordinate system].

One of the key signatures of the DPS is the large density of flat bands with modes of Gaussian tails. This can be used designing high quality broadband, and multichannel slow light devices. The anomalous dispersion of the DPS also can be engineered to generate new class of passive superluminal, and dispersion controlling devices. Although we have presented the DPS in one dimension for an optical wave, the idea can be easily extended, to generate harmonic oscillators, and therefore lossless effective dispersive metamaterials, in higher dimensions, and other physical wave systems.

## Methods

The continuous dispersion curve for the limit 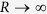, and the photonic band structures for finite values of *R* are obtained using the plane wave expansion method^29^. The continuous dispersion curve is obtained by increasing *R* to a huge value, until the results are converged. Specifically, in this paper we used *R* = 10000 to obtain the dispersion curve [Figs 2(a) and 4(d)]. The transmission spectrums for the DPS are obtained using transfer matrix method (TMM)^43^. In TMM simulations, we slice the continuous dielectric profile [Eqn. 1], into large number of spatial steps with uniform dielectric constants. We verified the TMM results independently, using the finite–difference time domain (FDTD)^44^ simulation. For FDTD simulation we used the freely available software, MEEP^45^.

## Additional Information

**How to cite this article**: Alagappan, G. and Png, C. E. Doubly Resonant Optical Periodic Structure. *Sci. Rep.* **6**, 20590; doi: 10.1038/srep20590 (2016).
